# Decreased Urinary Levels of *SIRT1* as Non-Invasive Biomarker of Early Renal Damage in Hypertension

**DOI:** 10.3390/ijms21176390

**Published:** 2020-09-02

**Authors:** Olga Martinez-Arroyo, Ana Ortega, Miriam Galera, Elena Solaz, Sergio Martinez-Hervas, Josep Redon, Raquel Cortes

**Affiliations:** 1 Cardiometabolic and Renal Risk Research Group, INCLIVA Biomedical Research Institute, 46010 Valencia, Spain; omartinez@incliva.es (O.M.-A.); migamar3@posgrado.upv.es (M.G.); elehigue@hotmail.com (E.S.); josep.redon@uv.es (J.R.); 2 Internal Medicine Unit, Hospital Clinico Universitario, 46010 Valencia, Spain; 3 Service of Endocrinology and Nutrition, Hospital Clinico Universitario, 46010 Valencia, Spain; sergio.martinez@uv.es; 4 CIBER of Diabetes and Associated Metabolic Diseases (CIBERDEM), Institute of Health Carlos III, Minister of Health, 28029 Madrid, Spain; 5 Department of Medicine, Faculty of Medicine and Odontology, University of Valencia, 46010 Valencia, Spain; 6 CIBER of Physiopathology of Obesity and Nutrition (CIBEROBN), Institute of Health Carlos III, Minister of Health, 28029 Madrid, Spain

**Keywords:** sirtuin 1, claudin 1, urinary albumin excretion, hypertension, diabetes mellitus, podocyte, miRNA

## Abstract

Sirtuins have become important players in renal damage in hypertension and diabetes, but their value as biomarkers is poorly assessed. The aims of the study were to evaluate the levels of sirtuin1 (SIRT1), and two miRNAs that regulate SIRT1 expression in hypertensive patients with incipient renal damage with and without diabetes. We quantified urinary *SIRT1* and claudin 1 (*CLDN1*) mRNA and miR34-a and miR-200a levels by quantitative real-time polymerase chain reaction (RT-qPCR) from patients and in cultured podocytes treated with high glucose and angiotensin II. Western blot and fluorescence analyses were also performed. We found decreased *SIRT1* levels in patients with increased urinary albumin excretion (UAE), the lowest with diabetes presence, and a strong association with UAE, discriminating incipient renal damage. In vitro experiments also showed SIRT1 overall decreases in podocyte cultures under treatment conditions. In urine samples, miR-34a was reduced and miR-200a increased, both related to UAE levels. However, both miRNAs were generally increased in podocyte cultures under high glucose and angiotensin-II treatment. These results show a significant urinary *SIRT1* decrease in albuminuric hypertensive patients, strongly associated with albuminuria, suggesting that *SIRT1* could be a potential and non-invasive method to assess incipient renal damage in hypertensive patients.

## 1. Introduction

Hypertension and diabetes mellitus (DM) are high prevalent diseases that often affect organ structure and microvasculature, as occur with the kidney, becoming leading causes of renal injury [1,2,3]. A progressive decrease in glomerular filtration rate (GFR) and/or an increase in urinary albumin excretion (UAE) are common features of renal damage [4,5]. In fact, UAE is a well-established prognostic marker in cardiovascular and renal diseases, widely used in clinical practice [6,7]. Nevertheless, using UAE measurement as a risk marker for the development of chronic kidney disease or end-stage renal disease remains controversial [8,9].

At structural level, during injury, the glomerulus and renal tubule are greatly compromised, subjected to morphological and molecular alterations [10,11,12]. Podocytes, highly differentiated cells, undergo changes and cell loss to the urine, which disturbs the glomerular filtration barrier, essential in preservation of renal function [11,13]. In addition, in tubular ducts tubule-interstitial inflammation and fibrosis are frequent [14,15]. Evaluation of lesions is invasively assessed by renal biopsy; however, alternative options have emerged based on the research for damage biomarkers in a patient’s urine [16,17,18].

Previous studies place the interest on the role of the NAD+-dependent deacetylases sirtuins, and concretely, sirtuin 1 (SIRT1) in renal pathologies [19]. Sirtuins regulate a wide range of biological pathways including energy metabolism, stress response, fibrosis or inflammation, through the induction of chromatin-silencing and transcriptional repression [20,21,22]. Decreases in SIRT1 have been reported in renal biopsies of diabetic patients and related with albuminuria [23]. Moreover, studies on animal models state a negative effect of podocyte *SIRT1* knockout (KO), with cytoskeleton rearrangements and worsening proteinuria [24,25], and *SIRT1* overexpression attenuates injury [26]. Our group has recently reported a strong association between the increased urinary levels of SIRT1 and histological features indicative of severity in lupus nephritis patients [27]. These studies support the relevance of the SIRT1 pathway in renal diseases, but its importance as disease marker in urine has not been addressed previously in DM and hypertension, the main leading causes of renal injury.

Studies report a close connection between tubule and podocyte in which SIRT1 and claudin 1 (CLDN1) are essential players [28]. Claudins are tight junction proteins that maintain corpuscular structure and determine paracellular permeability, expressed mainly in glomerular cells [29,30]. An influence of SIRT1 over *CLDN1* gene expression through epigenetic mechanisms has been described [23]. At the same time, gene expression controllers are susceptible to regulation, as occurs with *SIRT1* that has several microRNAs (miRNAs), such as miR-34a and miR-200a that targets its sequence to regulate expression [31].

In the present study, we aimed to analyse the levels of *SIRT1* and *CLDN1* in urine pellets from hypertensive patients with and without diabetes and/or albuminuria to unravel their potential value as non-invasive biomarkers of incipient renal damage. In addition, to mimic disease conditions and deepen into the implications of SIRT1 in podocyte injury, we have studied protein and mRNA changes in podocyte cultures subjected to high glucose (HG) and angiotensin II (Ang II) concentrations. Moreover, we have quantified miR-34a and miR-200a in both patients’ urine and in podocyte cultures to assess changes in *SIRT1* target miRNAs and if these changes are reflected in urine.

## 2. Results

### 2.1. Characteristics of Study Patients

Fifty-five hypertensive patients were included in the study. Of all, 25 were diagnosed with DM (18 with increased UAE, and 7 with normal UAE). The remaining 30 patients were non-diabetic (13 with increased UAE, 17 with normal UAE). Clinical characteristics of the study population are summarised in Table 1. For miRNA analyses, the study group was composed of 47 patients whose clinical characteristics are shown in Table S1.

### 2.2. Sirtuin 1 (SIRT1) and Claudin 1 (CLDN1) Levels in Urine from Hypertensive Patients

**Figure 1:**
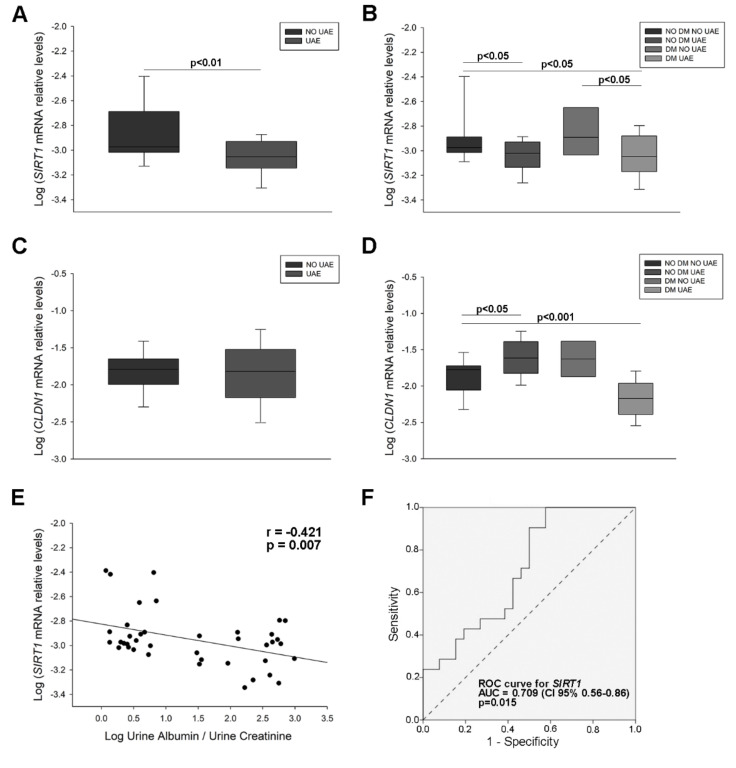
Sirtuin 1 (*SIRT1)* and Claudin 1 (*CLDN1)* mRNA levels in urinary sediment of hypertensive patients. (**A**) Box plot of the comparison of *SIRT1* mRNA levels between patients with increased UAE and normal UAE levels; (**B**) Box plot comparing *SIRT1* mRNA levels in patients with hypertension and normal UAE (NO DM NO UAE) with non-diabetic patients with increased UAE (NO DM UAE), diabetic patients with normal UAE (DM NO UAE) and diabetic patients with elevated UAE (DM UAE); (**C**) Box plot of the comparison of *CLDN1* mRNA levels between 2 groups (above described); (**D**) Box plot comparing *CLDN1* mRNA levels between patients divided in 4 groups (above described); (**E**) Scatter plot of the correlation between *SIRT1* mRNA levels and UAE values; (**F**) ROC curve analysis of *SIRT1* mRNA to test its predictive value for UAE discrimination. Horizontal bars represent median ± standard error of the mean (SEM). mRNA levels were normalised to two housekeeping genes and log relative expression was expressed: a gene is up-regulated when its relative values are higher in the disease group than those in controls. If the values are lower, the gene is down-regulated. The AUC and 95% CI are plotted in the graph. *SIRT1*: Sirtuin 1 gene; *CLDN1*: Claudin 1 gene; UAE: urinary albumin excretion; DM: diabetes mellitus; ROC: receiver operating characteristic; AUC: area under the curve; CI: confidence interval.

*SIRT1* mRNA levels were significantly decreased in patients with increased UAE compared to non-albuminuric patients (1.66-fold decrease, *p* < 0.01) (Figure 1A). Dividing the study group according to diabetic disease and increased albuminuria showed also lower levels in non-diabetic and diabetic patients with increased UAE as compared with those with normal UAE values (1.64-fold and 1.77-fold decrease, respectively, *p* < 0.05 for both) (Figure 1B). *CLDN1* mRNA levels were higher in the non-diabetic group with increased UAE and lower in the diabetic group with increased UAE (1.87-fold increase, *p* < 0.05 and 1.90-fold decrease, *p* < 0.001), (Figure 1C,D). Finally, we observed an inverse correlation between *SIRT1* mRNA levels and UAE (r = −0.421, *p* = 0.007), (Figure 1E). A receiver operating characteristic (ROC) curve constructed for *SIRT1* mRNA to evaluate its diagnostic value in predicting the presence of UAE revealed an area under the curve (AUC) = 0.709 (95% confidence interval (CI) 0.56–0.86; *p* = 0.015) (Figure 1F). These results evidence the presence of significant decreases of *SIRT1* and increases of *CLDN1* in urine from hypertensive patients with increased UAE and a strong relationship of *SIRT1* with albuminuria.

### 2.3. SIRT1 and CLDN1 Levels in Podocyte Cultures Subjected to Stress

**Figure 2:**
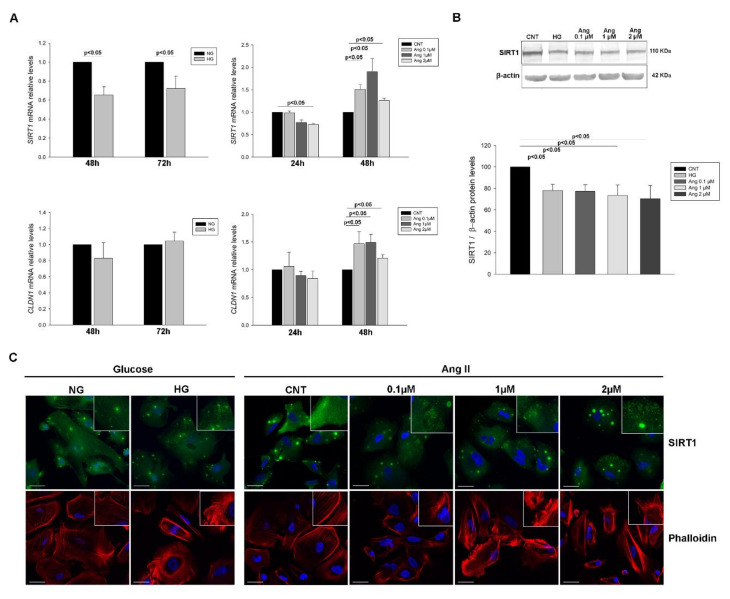
*SIRT1* and *CLDN1* levels in podocyte cultures subjected to HG and Ang II treatments. (**A**) Bar graphs of the comparison of *SIRT1* and *CLDN1* mRNA levels between NG and HG and between Ang increasing concentrations at different treatment times; (**B**) Blots of SIRT1 protein levels in glucose and Ang II treated podocytes; (**C**) Immunofluorescence analyses of SIRT1 in treated podocytes and phalloidin staining of F-actin fibers showing the cytoskeleton dysregulation under treatments. Bars represent mean ± SEM (*n* = 6 each group). mRNA levels were normalised to two housekeeping genes and relative expression was expressed in fold change with CNT group values set to 1-fold. Protein levels were previously normalised to β-actin and expressed as arbitrary units, with CNT values set to 100. *SIRT1*: Sirtuin 1 gene; *CLDN1*: Claudin 1 gene; NG: normal glucose; HG: high glucose; CNT: control; Ang: angiotensin II. Scale bar: 40 µm.

The treatment of podocytes with HG and increasing concentrations of Ang II at different treatment times showed reduced levels of *SIRT1* mRNA in HG group (1.53-fold and 1.38-fold decrease at 48 h and 72 h post-treatment, respectively, *p* < 0.05) and at 24 h treatment with Ang II 2 µM (1.37-fold decrease, *p* < 0.01), while at 48 h levels increased in all concentrations (1.51-fold, 1.90-fold and 1.26-fold increase, respectively, *p* < 0.05 in all). *CLDN1* mRNA levels remained unchanged with HG treatment but increased with Ang II at 48 h treatment (1.47-fold (0.1 µM), 1.49-fold (1 µM) and 1.21-fold increase (2 µM), *p* < 0.05 in all), (Figure 2A).

Protein levels of SIRT1 were reduced under HG (1.28-fold decrease, *p* < 0.01) in concordance to mRNA levels, and in all Ang II concentrations assayed (1.29-fold, 1.36-fold and 1.42-fold decrease, *p* < 0.05 in all) (Figure 2B). CLDN1 protein did not show significant changes in glucose and Ang II treatments, only a slight increase trend (Figure S1). Immunofluorescence analysis of SIRT1 showed both nucleus and cytoplasm protein localization with protein accumulations and evidenced decreased fluorescence intensity and a more granulated cell pattern distribution under both treatments. Moreover, analysis of F-actin with phalloidin staining revealed a disruption of cytoskeleton under these treatments (Figure 2C and Figure S2). These findings show an overall decrease in both mRNA and protein SIRT1 levels in podocytes subjected to stress.

### 2.4. MiR-34a-and miR-200a Levels in Urinary Sediment and Podocyte Culture Pellets

#### 2.4.1. MiRNA Levels in Urinary Sediment from Hypertensive Patients

**Figure 3:**
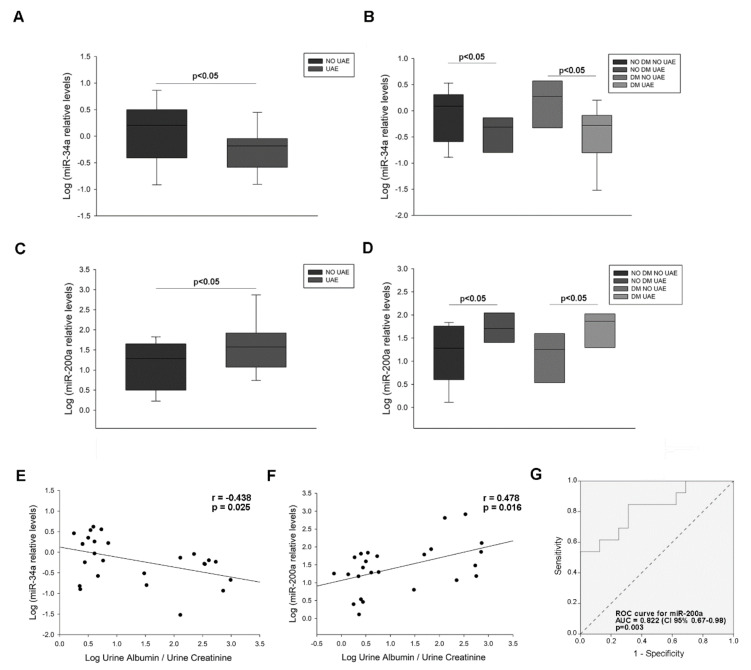
miRNAs levels in urinary sediment of hypertensive patients. (**A**) Box plot of the comparison of miR-34a levels between patients with increased UAE and normal UAE levels; (**B**) Box plot comparing miR-34a levels in patients with hypertension and normal UAE (NO DM NO UAE) with non-diabetic patients with increased UAE (NO DM UAE), diabetic patients with normal UAE (DM NO UAE) and diabetic patients with elevated UAE (DM UAE); (**C**) Box plot of the comparison of miR-200a levels between 2 groups (above described); (**D**) Box plot comparing miR-200a levels between patients divided in 4 groups (above described); (**E**) Scatter plot of the correlation between miR-34a levels and UAE values; (**F**) Scatter plot of the correlation between miR-200a levels and UAE values; (**G**) ROC curve analysis of miR-200a to test its predictive value for UAE discrimination. Horizontal bars represent median ± SEM. miRNA levels were normalised to two housekeeping miRNAs and log relative expression was expressed: a miRNA is up-regulated when its relative values are higher in the disease group than those in controls. If the values are lower, the miRNA is down-regulated. The AUC and 95% CI are plotted in the graph. UAE: urinary albumin excretion; DM: diabetes mellitus; ROC: Receiver operating characteristic; AUC: area under the curve; CI: confidence interval.

MiRNA levels of miR-34a and miR-200a were measured in patient’s urine and podocyte culture pellets. MiR-34a showed decreased levels in albuminuric patients compared to non-albuminuric (5.01-fold decrease, *p* < 0.05) (Figure 3A), and also analysed by diabetic and albuminuric groups, showing a 2.97-fold decrease (*p* < 0.05) the non-diabetic group with albuminuria, and 2.31-fold decrease (*p* < 0.05) the diabetic and albuminuric group (Figure 3B). On the other hand, miR-200a was increased in albuminuric patients (6.04-fold increase, *p* < 0.05), and also in the diabetic group with albuminuria (2.41-fold increase, *p* < 0.05) (Figure 3C). Levels of miR-34a and miR-200a were correlated with UAE (r = −0.438, *p* = 0.025 and r = 0.478, *p* = 0.016, respectively) (Figure 3E,F). ROC curves constructed for miR-200a revealed an AUC = 0.822 (95% CI 0.67–0.98; *p* = 0.003) (Figure 1G). These analyses show that miR-34a is reduced and miR-200a increased in patients with elevated UAE and a relationship of both miRNAs with albuminuria.

#### 2.4.2. MiRNA Levels in Treated Podocyte Cultures

**Figure 4:**
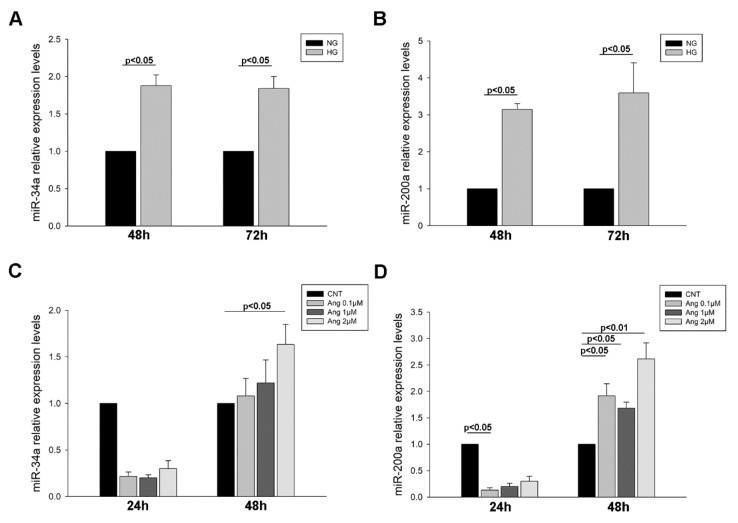
miRNAs levels in podocyte cultures subjected to HG and Ang II treatments. (**A**) Bar graphs of the comparison of miR-34a levels between NG and HG treated podocytes; (**B**) Bar graphs of the comparison of miR-200a levels between NG and HG treated podocytes; (**C**) Bar graphs of the comparison of miR-34a levels between Ang increasing concentrations; (**D**) Bar graphs of the comparison of miR-200a levels between Ang increasing concentrations. Bars represent mean ± SEM (*n* = 6 each group). miRNA levels were normalised to two housekeeping miRNAs and relative expression was expressed in fold change with CNT group values set to 1-fold. NG: normal glucose; HG: high glucose; CNT: control; Ang: angiotensin II.

Podocytes treated with HG showed an increase in miR-34a levels at both time of exposure to treatment (1.88-fold increase (48 h) and 1.84-fold increase (72 h), *p* < 0.05 for both) (Figure 4A). The same was observed for miR-200a (3.15-fold increase (48 h) and 3.59-fold increase (72 h), *p* < 0.05 for both) (Figure 4B). When treated with increasing concentrations of Ang II, miR-34a levels showed a decreasing trend at 24 h treatment but at 48 h and 2 µM concentration its levels augmented (1.63-fold increase, *p* < 0.05) (Figure 4C). A similar effect was observed with miR-200a levels, with decreases at 24 h Ang II treatment (7.47-fold decrease, *p* < 0.05) and increases at 48 h (1.92-fold (0.1 µM, *p* < 0.05), 1.68-fold (1 µM, *p* < 0.05) and 2.62-fold increase (2 µM, *p* < 0.01)) (Figure 4D). Taken together, these results evidence that both miRNAs are increased under the HG condition and when treated with Ang II their levels change depending on treatment time.

## 3. Discussion

This is the first study that measures urinary *SIRT1* and *CLDN1* in hypertensive patients with or without diabetic condition and/or increased albuminuria. The analysis shows how decreased levels of *SIRT1* in diseased patients are inversely correlated to UAE levels. In addition, we observed this reduction in human podocyte cultures placed in HG milieu and Ang II treatments. Finally, the levels of miR-34a and miR-200a, regulators of *SIRT1* gene expression, show association with increased UAE levels and distinct alterations depending on treatment duration in podocytes.

It is worthy of note that the implication and importance of SIRT1 in kidney-related diseases, such as diabetic nephropathy [26,32,33,34], acute and chronic kidney disease [19,35,36] or lupus nephritis [27,37] can be explained by its involvement in numerous pathways regulation. SIRT1 regulates the TGFβ/Smad and ERK1/2 pathways, also activates metalloproteinases and FOXO1 proteins and controls the NF-kB pathway [24,37,38]. These routes are activated or inhibited when SIRT1 expression decreases, which leads to an increase in cell apoptosis, fibrosis, inflammation and oxidative stress. With this relevance of SIRT1 controlling a wide range of pathways, previous studies highlight the potential therapeutic role of *SIRT1* activation/overexpression in attenuating injury [35,39], suggesting an important function of decreased SIRT1 in albuminuria and renal function decline. In light of these data, we have also observed marked decreases in both patients with albuminuria and stressed podocyte cultures that reinforces the SIRT1 role in kidney disease progression.

In this study, the analyses performed on patients have revealed a decreased expression of *SIRT1* mRNA in urinary sediment from hypertensive subjects with increased UAE and also in the hypertensive and diabetic group with elevated UAE. These findings further support the role of SIRT1 in renal pathology, independent of the causal factor, such as hypertension or diabetes. Moreover, this decrease on urine reflects the reduced levels found in renal biopsies of diabetic nephropathy patients, in which the decline was observed prior to albuminuria development [23] and also in fibrotic kidney from focal glomerulosclerosis patients [38]. Additionally, we have found an inverse correlation between urinary *SIRT1* mRNA and UAE levels, and the ROC curve analysis showed that *SIRT1* mRNA measured on urine may discriminate the presence of elevated UAE in patients. Renal biopsy procedure is an invasive method for renal injury detection, and is an unusual practice in hypertensive and diabetic nephropathy, so there is a need for finding non-invasive alternatives. In this context, we could place *SIRT1* urine mRNA quantification as non-invasive method to indicate the presence of an incipient renal damage.

Animal model studies report reduced levels of SIRT1 in tubule and podocytes. SIRT1 overexpression and KO models revealed prevention and aggravation of the glomerular changes and established a cross-talk between these renal portions, founding an influence of tubule SIRT1 on podocyte SIRT1 levels [23,25,26]. In line with this, we have found this reduction in both mRNA and protein SIRT1 levels of treated podocytes, without having the influence of tubular SIRT1 levels. This evidences that a stress induction such as HG and Ang II is enough to alter SIRT1 in the podocyte, as observed recently in glucose overload treatments on podocytes [40,41]. Strikingly, Ang II treatment produced an opposite effect on *SIRT1* mRNA expression, which was decreased at 24 h treatment and increased at prolonged exposure time. Related studies found also a decrease of mRNA and protein SIRT1 levels at 24 h treatment, but no longer time treatments were assayed [42]. Taking these results together, we think there could be an opposed influence of Ang II on SIRT1 expression over time or maybe there are other factors influencing on its levels, as discussed below regarding miRNAs implication. Furthermore, the analyses of *SIRT1* in patient’s urine seem to reflect the decreases found in podocytes.

On the other hand, tight junction proteins such as CLDN1 are visibly the main counterparts of glomerular structure and function [29]. In fact, high levels of CLDN1 have been reported on podocytes from diabetic models, leading to podocyte effacement and albuminuria [23]. In addition, upregulation of *CLDN1* in murine podocytes causes slit diaphragm-tight junction destabilization and consequently proteinuria [43]. Going further, the existence of a close communication between proximal tubule-podocyte, places tubular SIRT1 as an epigenetic regulator of *CLDN1* expression in podocytes, since SIRT1 downregulates the *CLDN1* gene by deacetylating histone H3 and H4 [23,28]. The dysfunction of this cross-talk in the kidney precedes malfunctioning and podocyte injury [44]. Maybe because of this influence, we could not observe changes in CLDN1 levels in our in vitro experiments with glucose-overloaded podocytes, although we found increases after the longest treatment with Ang II. Studies on podocyte cultures are coincident with this observation, and levels only changed when incubated with conditioned medium from tubular cells [23]. Overall, these results could indicate the need of this retrograde interplay between tubule and glomerulus to effectively influence on CLDN1 levels, since SIRT1 diminution in podocytes may be not enough for this regulation.

Just as some biological or synthetic compounds have been revealed as activators of *SIRT1* expression, special attention could be focused on the regulation through miRNAs, hence *SIRT1* activation to reverse injury could be directed by its target miRNAs. There is not a specific miRNA regulating one mRNA, and in the case of *SIRT1*, there are several described to target its mRNA sequence, as miR-34a and miR-200a [31,33]. In the present research, we have quantified both miRNAs’ levels in urinary sediment from patients and in treated podocytes. MiRNA-34a showed decreased levels and miR-200a increased in urine samples from albuminuric patients with or without diabetes, while in treated podocytes we observed for both miRNAs an increase in HG group and different directions depending on Ang II treatment time. In the context of renal diseases, particularly in diabetic nephropathy, miR-34 has shown increased levels in mesangial and tubular cells treated with HG and was also increased in diabetic nephropathy mice and patients; these changes in tubular cells were attributed to *SIRT1* regulation by miR-34a [45,46,47]. Our finding in HG-treated podocytes sustains these previous observations. In addition, increases in circulating miR-34a showed associations with a higher clinical stage of hypertension [48], but was found to be reduced in a hypertensive model, and it was proposed that its overexpression was a beneficial target for improving renal function in this disease [49]. Concerning miR-200a, its involvement in renal diseases through *SIRT1* regulation has not been reported to date. However, its increased levels in urine have been proposed as a diagnostic tool for kidney injury [50,51], while contrarily its downregulation has been suggested to promote renal fibrosis in vitro [52]. Regarding our results, the elevated levels in patient’s urine and HG-treated podocytes, and the decreases in SIRT1 observed, could suggest that miR-200a could be regulating *SIRT1* in podocytes, at least in glucose milieu. Moreover, our findings showed a direct relationship between miR-200a levels and UAE levels and the ROC curve analysis establish that this miRNA is capable of discriminate the presence of elevated UAE in patients. Nonetheless, future studies should be directed to ascertain this particular role of miR-200a and SIRT1 and the mechanisms responsible for this interaction in kidney damage.

As shown also in our Ang II-treated podocytes, there seems to be a controversial effect on both miRNAs depending on treatment times that will require further investigation. Nevertheless, our results shed some light into the involvement of miR-34a in diabetes and hypertension. Urinary levels may reflect how this miRNA alters its expression in the presence of hypertension, since these levels are also reduced in patients without diabetic disease, being coincident with the observed in the hypertensive models [49].

Finally, although both miRNAs have been reported to suppress *SIRT1* levels, there is an open question about the variety of functions of miRNAs as genetic regulators. Recent findings place novel functions for miRNAs as transcriptional regulators by returning to the nucleus and exerting a gene transcriptional activation or silencing [53,54]. Hence, we could propose that the influence of both miRNAs on *SIRT1* expression could be different depending on the disease context and that obviously other factors could be influencing this regulation, acting as a whole regulatory mechanism in disease. However, further experiments are needed to explore how these miRNAs influence SIRT1.

Collectively these results evidence a reduction of *SIRT1* in urine from hypertensive patients with or without a diabetic condition. Furthermore, the decrease of SIRT1 and increase of CLDN1 in patients and podocytes subjected to stress support the implication of both in renal injury and evidence a reflection in urinary levels. The alterations found in miR-34a and miR-200a both in patients’ urine and podocyte cultures reveal novel points to further address their implication in renal injury through *SIRT1* regulation. Interestingly, SIRT1 mRNA levels and miR-200a are related to increases in UAE and discriminate the presence of UAE in patients. In summary, our results point out urinary *SIRT1* mRNA measurements as an easily accessible and non-invasive method to early characterize renal damage in patients.

## 4. Materials and Methods

### 4.1. Study Population

The study included 55 hypertensive patients with and without DM, from the Internal Medicine area of Hospital Clínico Universitario of Valencia (Spain). Hypertension was defined according to the European Society of Hypertension (systolic blood pressure (SBP) > 140 mmHg and/or diastolic blood pressure (DBP) < 90 mmHg) [55], and DM according to the World Health Organization [56]. Among all patients, 31 showed increased UAE. All patients received antihypertensive treatment and DM subjects were treated with oral antidiabetic agents. All patients gave written informed consent to participate in the study. The study protocol was approved by the Ethics Committee of the Hospital Clínico Universitario of Valencia (N° 2016/084 (19 May 2016)) in accordance with the Declaration of Helsinki of 1975 as revised in 2013 [57].

### 4.2. Urine Samples Processing and Urinary Albumin Excretion (UAE) Data Measurements

Fresh urine samples were obtained from the first morning urine and were centrifuged at 2250× *g* for 30 min at 4 °C. Cell pellet was washed with 1 mL of sterile phosphate-buffered saline (PBS), and centrifuged at 15,000× *g* for 5 min at 4 °C, obtaining a clean cell pellet. UAE was measured using a nephelometric immunoassay (Behring nephelometer). For each patient, albuminuria was considered as the mean value obtained from the morning spot urine samples and expressed as albumin (mg)/creatinine (g) ratio (ACR). Increased UAE was defined as ACR ≥ 30 mg/g.

### 4.3. Human Podocytes Culture and Treatment

Cell line AB8/13 of conditionally human immortalised podocytes was kindly provided by Prof Moin Saleem (Children’s Renal Unit and Academic Renal Unit, University of Bristol, Southmead Hospital, Bristol, UK) [58]. Briefly, podocytes were cultured in RPMI 1640 with 10% foetal bovine serum (FBS) (Biowest, Nuaillé, France), 1% insulin-transferrin-selenium (ITS) (Gibco, Thermo-Fisher Scientific, Waltham, MA, USA) and 1% penicillin/streptomycin. Cells were propagated until 80% confluence at 33 °C and replated at proper density. Next, podocytes were differentiated at 37 °C for 10–14 days with lower FBS concentration (2%). All experiments were performed on differentiated podocytes. Cells were serum starved and then treated with normal (NG) (5.5 mM d-Glucose) and HG (30 mM d-Glucose) concentrations for 48 h and 72 h. Also, cells were treated with Ang II at 0.1, 1 and 2 µM for 24 h and 48 h.

### 4.4. RNA Isolation and cDNA Synthesis

Total RNA enriched with miRNA was extracted from urine samples and podocyte pellets using the miRNeasy mini kit (Qiagen, Hilden, Germany) following the manufacturer’s instructions. RNA concentrations and purity were assessed by NanoDrop 2000 spectrophotometer (ThermoFisher Scientific, Waltham, MA, USA) and samples were stored at −80 °C until use. After, for mRNA analyses, RNA was reverse-transcribed to cDNA using the kit Ready-To-Go You-Prime First-Strand Beads (GE Healthcare, Buckinghamshire, UK) following the protocol indications. For miRNA analysis, TaqMan™ Advanced miRNA cDNA Synthesis Kit (Applied Biosystems, Foster City, CA, USA) was used to prepare the cDNA template from 2 μL of total RNA. For all analyses, the obtained cDNA was stored at −80 °C until use.

### 4.5. Quantitative Real-Time Polymerase Chain Reaction (RT-qPCR)

Quantitative real time PCR (RT-qPCR) experiments were performed in the LightCycler 480 II real-time PCR system (Roche, Mannheim, Germany). Regarding mRNA analyses, Qiagen Multiplex PCR Master Mix with LC Green reagent (Qiagen, Hilden, Germany) and specific primers were used. For miRNA analyses, 2.5 μL of diluted cDNA was combined with TaqMan^®^ Fast Advanced Master Mix (2X), and specific TaqMan™ Advanced microRNA assay probes (Applied Biosystems, Foster City, CA, USA) for miR-34a-5p (ID: 478048) and miR-200a-3p (ID: 478490). Changes in mRNA and miRNA levels were analysed by absolute quantification as previously described [27]. Briefly, standard curves for each target gene or miRNA were performed to calculate the absolute number of copies. Concentrations were calibrated from 1 × 10^9^ to 1 copy number per microgram of cDNA by serial 10-fold dilutions. This known concentrations and copy numbers of target genes and miRNAs were assayed in the qPCR experiments joint to experimental samples and were normalised to *ACTB* and *B2M* housekeeping genes for mRNA analyses and to miR125a-5p (ID: 477884) and miR186-5p (ID: 477940) for miRNA analyses. In the mRNA levels analysis, primer design of target genes for standard curves and RT-qPCR was carried out with Primer 3 software (Table 2), and melting curve analysis was performed to evaluate the specificity of the amplicons generated. All assays were run in triplicate, including appropriate controls and the non-template control. The values for each analysed gene and miRNA were expressed as the log ratio or fold change between the target gene and the average value of housekeeping genes.

### 4.6. Homogenization of Samples, Electrophoresis and Western Blot

Urine samples and podocyte cell pellets were lysed using radioimmunoprecipitation assay (RIPA) buffer (ThermoFisher Scientific, Waltham MA, USA) with protease inhibitor cocktail (Sigma-Aldrich, St Louis, MO, USA). Samples were centrifuged at 15,000× *g* for 15 min at 4 °C and supernatant was collected. Protein quantification was performed by Lowry method using bovine serum albumin (BSA) as standard. Protein homogenates were loaded into NuPAGE 4–12% polyacrylamide gels or NuPAGE 3–8% tris-acetate gels (Invitrogen, Carlsbad, CA, USA) and were then transferred to polyvinylidene difluoride (PVDF) membranes for Western blotting. After blocking, the membranes were incubated with primary antibodies at room temperature (RT) for 2 h. The primary antibodies used were rabbit monoclonal anti-sirt-1 (1/3000, Abcam, Cambridge, UK), rabbit monoclonal anti-claudin-1 (1/2000, Abcam, Cambridge, UK). Monoclonal anti-β-actin antibody (1/6000, Sigma-Aldrich, St Louis, MO, USA) was used as loading control. Then, membranes were washed 3 times with tris-buffered saline with Tween 20 (TBS-T, 20  mM tris-HCl, 150  mM NaCl and 0.1% Tween 20) and incubated with alkaline phosphatase-conjugated anti-rabbit IgG or anti-mouse IgG antibodies (Sigma-Aldrich, St Louis, MO, USA) at RT for 1  h. After, they were washed 3 times with TBS-T and TBS and the chromogen 5-bromo-4-chloro-3-indolyl phosphate/nitro blue tetrazolium (BCIP/NBT, Sigma-Aldrich, St Louis, MO, USA) was used to detect bound antibodies. Finally, bands were visualised using an imaging system and quantified by the TotalLab TL-100 (v.2008) program.

### 4.7. Immunofluorescence Analyses

Analysis by fluorescence was performed using a Leica confocal microscope DMi8 (Leica Microsystems, Wetzlar, Germany) and images were processed with ImageJ (v. 1.46 r; National Institutes of Health, Bethesda, MD, USA) software. For this, samples were processed as in [59] and incubated with anti-sirt-1 (1/100). F-actin cytoskeleton distribution was evaluated by phalloidin staining on treated podocytes with HG and Ang II concentrations. Cells were fixed in 4% paraformaldehyde for 15 min at room temperature (RT), washed three times with PBS, permeabilised with 0.1% Triton X-100, washed and incubated with Phalloidin-iFluor 594 Reagent (1/1000, Abcam, Cambridge, UK) for 1 h at RT. Within the last half hour of the incubation period, cells were stained with DAPI. After incubation, cells were washed 3 times in PBS, mounted and observed.

### 4.8. Statistical Analyses

Data are expressed as fold change and mean ± standard error of the mean (SEM). The Kolmogorov–Smirnov test was used to analyse the normal distribution of the variables. Comparisons between two groups were performed with Student’s *t*-test or Mann–Whitney U-test depending on data distribution, and Pearson’s correlation coefficient was calculated to analyse the association between variables. The sensitivity, specificity and predictive value of *SIRT1* mRNA and miR-200a was calculated by generating ROC curves, with calculation of the AUC and with 95% confidence interval (CI). The significance level was set at *p* < 0.05. Statistical analyses were performed with the SPSS Software v. 20 (IBM SPSS Inc., Chicago, IL, USA) and graphics were created with SigmaPlot™ (Systat Software, Inc., San Jose, CA, USA).

## Figures and Tables

**Table 1:** Clinical characteristics of hypertensive patients divided in study groups.

	Non-Diabetic		Diabetic	
Variables	Increased UAE (*n* = 13)	No UAE (*n* = 17)	Increased UAE (*n* = 18)	No UAE (*n* = 7)
**Age (Years)**	50.38 ± 9.52 ††	54.94 ± 5.89	60.00 ± 7.69 ‡‡	55.00 ± 4.73
**Gender (Male)**	61.5%	58.8%	72.2%	71.4% §
**BMI (kg/m^2^)**	29.47 ± 5.21	30.33 ± 5.80	34.16 ± 7.41	28.11 ± 4.30
**SBP (mmHg)**	130.08 ± 10.67	132.88 ± 18.08	140.89 ± 25.77	143.71 ± 38.75
**DBP (mmHg)**	81.85 ± 8.86	87.47 ± 11.65	82.00 ± 12.89	92.14 ± 16.89
**Glucose (mg/dL)**	97.77 ± 18.12 †††	103.18 ± 9.93	151.33 ± 56.22 ‡‡	154.14 ± 67.59 §
**Glycated Hb (%)**	5.77 ± 0.07 †††	5.63 ± 0.21	6.99 ± 1.09 ‡‡‡	6.46 ± 1.17
**T Cholesterol (mg/dL)**	207.38 ± 36.14 †	185.05 ± 24.34	185.17 ± 32.66 *	154.29 ± 20.87 §§
**LDL (mg/dL)**	135.31 ± 30.49 †	117.88 ± 20.01	115.28 ± 28.46 *	90.71 ± 18.9 §§
**HDL (mg/dL)**	57.00 ± 13.82 ††	51.76 ± 10.82	43.56 ± 11.02 ‡‡	46.00 ± 8.32
**TG (mg/dL)**	111.38 ± 44.90 ††	118.71 ± 59.64	212.33 ± 142.66 ‡‡	140.43 ± 49.61
**Plasma Cr (mg/dL)**	0.92 ± 0.35	0.86 ± 0.18	0.95 ± 0.31	0.92 ± 0.25
**GFR (mL/min/1.73 m^2^)**	91.69 ± 30.44	88.01 ± 17.06	90.02 ± 25.96	87.61 ± 23.85
**Ratio UAE/Cr (mg/g)**	131.44 ± 210.89 †††	4.72 ± 6.78	343.18 ± 259.06 **‡‡	3.40 ± 1.36

BMI: body mass index; Cr: creatinine; DBP: diastolic blood pressure; GFR: glomerular filtration rate; Hb: haemoglobin; HDL: High-density lipoprotein; LDL: Low-density lipoprotein; SBP: systolic blood pressure; T Cholesterol: total cholesterol; TG: triglycerides; UAE: urinary albumin excretion. Comparisons between diabetic groups: * *p* < 0.05, ** *p* < 0.01. Comparisons between diabetic and non-diabetic groups: † *p* < 0.05; †† *p* < 0.01; ††† *p* < 0.001. Comparisons between increased UAE groups: ‡ *p* < 0.05, ‡‡ *p* < 0.01, ‡‡‡ *p* < 0.001. Comparisons between No UAE groups: § *p* < 0.05, §§ *p* < 0.01.

**Table 2:** Primer sequences for real-time polymerase chain reaction (RT-qPCR) analysis.

Target (Gene Name)	Primer	Sequence 5′→3′	Size, bp
*SIRT1*	Std-curve-F	agctgatgaaccgcttgctat	300
	Std-curve-R	ttggcatattcaccacctaacc	
	qPCR-F	ttgttattgggtcttccctcaaa	112
	qPCR-R	aaatgcagatgaggcaaaggtt	
*CLDN1*	Std-curve-F	agcacattgcaagcaacccgtgcct	320
	Std-curve-R	agggcacctcccagaaggcagaga	
	qPCR-F	ccgttggcatgaagtgtatg	101
	qPCR-R	agccagacctgcaagaagaa	
*ACTB*	Std-curve-F	gaggcatcctcaccctgaagta	232
	Std-curve-R	acagcctggatagcaacgtaca	
	qPCR-F	tggagaaaatctggcaccac	125
	qPCR-R	catgatctgggtcatcttctcg	
*B2M*	Std-curve-F	ctactctctctttctggcctggag	511
	Std-curve-R	aaacatggagacagcactcaaagt	
	qPCR-F	tccagcgtactccaaagattc	113
	qPCR-R	gtcaacttcaatgtcggatgg	

*SIRT1*: Sirtuin 1 gene; *CLDN1*: Claudin 1 gene; *ACTB*: actin β gene; *B2M*: β-2-microglobulin gene; Std-curve-F/R: Standard curve forward or reverse primer; qPCR-F/R: quantitative real time PCR forward or reverse primer.

## Supplementary Materials

Supplementary materials can be found at https://www.mdpi.com/1422-0067/21/17/6390/s1. Table S1: Clinical characteristics of hypertensive patients included in miRNA analysis. Figure S1: CLDN1 protein levels in podocyte cultures subjected to HG and ANG II treatments. Figure S2: Immunofluorescence magnifications of SIRT1 and F-actin staining in podocytes subjected to HG and Ang II treatments.

## Abbreviations

Ang II	Angiotensin II
AUC	Area under the curve
CLDN1	Claudin 1
DM	Diabetes mellitus
GFR	Glomerular Filtration Rate
HG	High Glucose
NG	Normal Glucose
ROC	Receiver operating characteristic
SIRT1	Sirtuin 1
UAE	Urinary Albumin Excretion
