# Supplementary material for: Decreased Urinary Levels of SIRT1 as Non-Invasive Biomarker of Early Renal Damage in Hypertension
Source: Int J Mol Sci. 2020 Sep 2;21(17):6390. doi: 10.3390/ijms21176390 (PMC7503821; doi:10.3390/ijms21176390)
Supplement: Supplementary file 1 [file ijms-21-06390-s001.zip › Figure S1.docx]

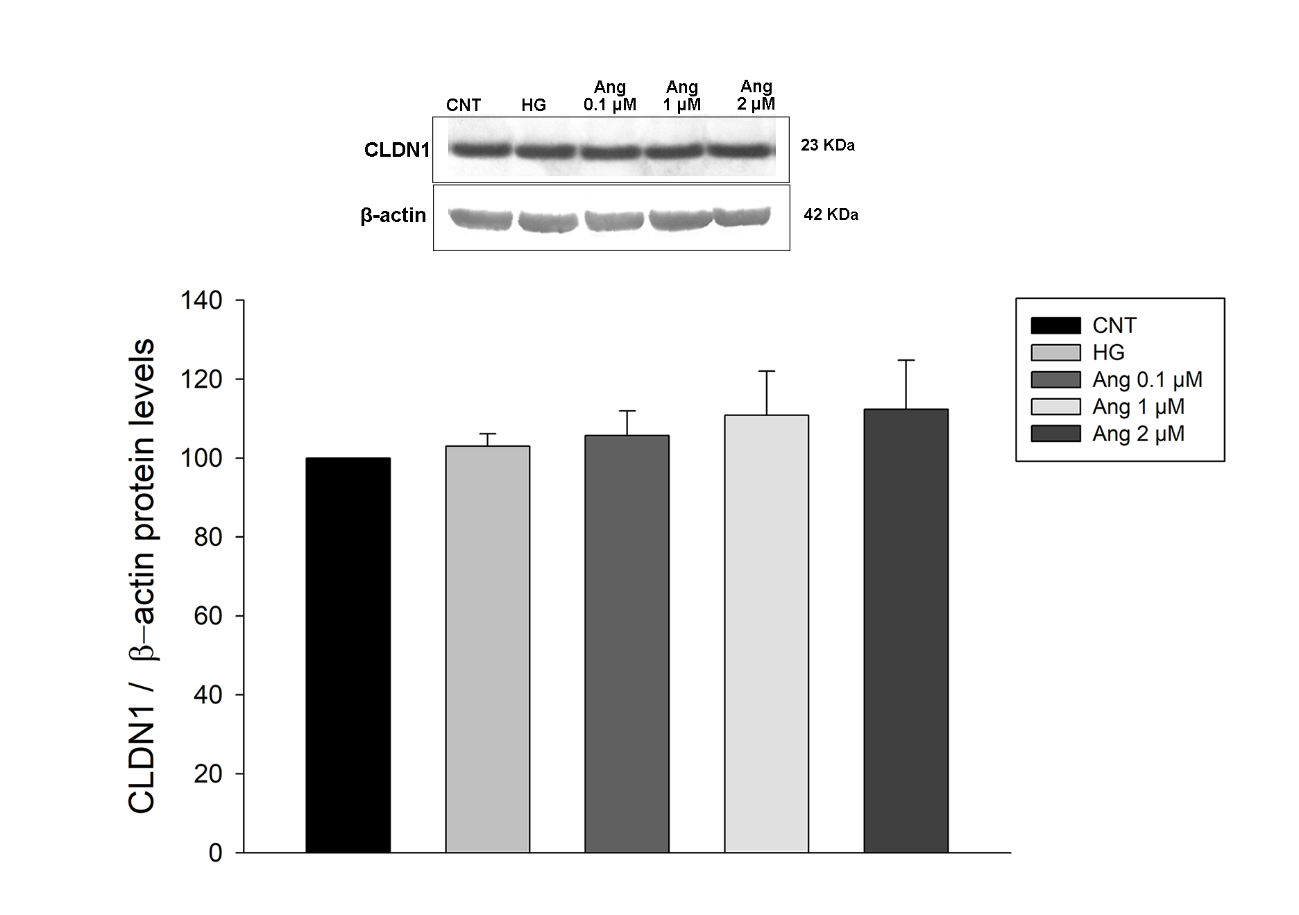


**Figure 1.** CLDN1 protein levels in podocyte cultures subjected to HG and Ang II treatments. Bars represent mean ± SEM (n = 6 each group). Protein levels were previously normalised to β-actin and expressed as arbitrary units, with CNT values set to 100. CLDN1: Claudin 1; NG: normal glucose; HG: high glucose; CNT: control; Ang: angiotensin II.
