# Supplementary material for: Decreased Urinary Levels of SIRT1 as Non-Invasive Biomarker of Early Renal Damage in Hypertension
Source: Int J Mol Sci. 2020 Sep 2;21(17):6390. doi: 10.3390/ijms21176390 (PMC7503821; doi:10.3390/ijms21176390)
Supplement: Supplementary file 1 [file ijms-21-06390-s001.zip › Figure S2.docx]

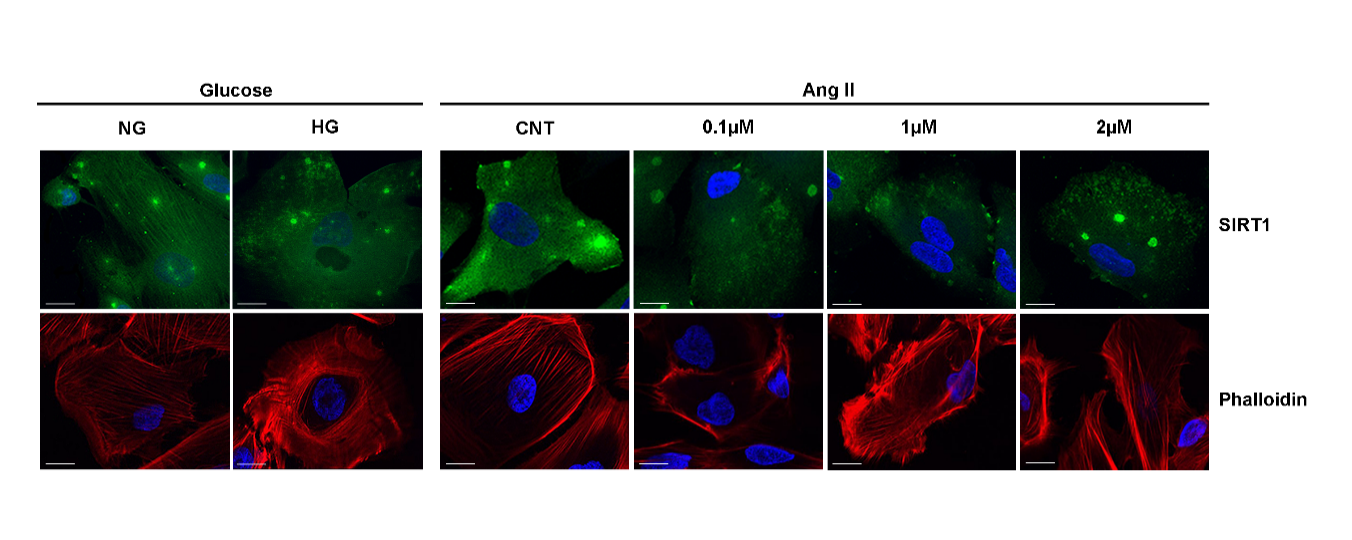


**Figure S2.** Immunofluorescence magnifications of SIRT1 and F-actin staining in podocytes subjected to HG and Ang II treatments. Bars represent mean ± SEM (n = 6 each group). *SIRT1*: Sirtuin 1; NG: normal glucose; HG: high glucose; CNT: control; Ang: angiotensin II. Scale bar: 20 µm.
