# Supplementary material for: Decreased Urinary Levels of SIRT1 as Non-Invasive Biomarker of Early Renal Damage in Hypertension
Source: Int J Mol Sci. 2020 Sep 2;21(17):6390. doi: 10.3390/ijms21176390 (PMC7503821; doi:10.3390/ijms21176390)
Supplement: Supplementary file 1 [file ijms-21-06390-s001.zip › Table S1.docx]

**Table S1.** Clinical characteristics of hypertensive patients included in miRNA analysis.

|  | Non-Diabetic | | | Diabetic | |
| --- | --- | --- | --- | --- | --- |
| Variables | **Increased UAE**  **(*n* = 10)** | **No UAE**  **(*n* = 15)** | **Increased UAE**  **(*n* = 13)** | | **No UAE**  **(*n* = 9)** |
| Age (Years) | 51.10 ± 11.03† | 54.07 ± 6.32 | 60.54 ± 10.16‡ | | 54.78 ± 4.15 |
| Gender (Male) | 40% | 53.3% | 30.8% | | 22.2%§ |
| BMI (kg/m^2^) | 27.65 ± 3.96† | 30.28 ± 7.04 | 35.81 ± 7.56*‡‡ | | 29.51 ± 4.66 |
| SBP (mmHg) | 129.90 ± 9.02† | 132.47 ± 19.98 | 147.54 ± 22.81‡§ | | 140.78 ± 34.06 |
| DBP (mmHg) | 83.00 ± 10.25 | 88.87 ± 13.75 | 85.92 ± 12.49 | | 89.00 ± 18.04 |
| Glucose (mg/dL) | 92.60 ± 13.85††† | 103.40 ± 7.84 | 153.54 ± 52.70‡‡‡ | | 149.11 ± 59.53§§ |
| Glycated Hb (%) | 5.7 ± 0.01††† | 5.59 ± 0.20 | 6.98 ± 1.35‡‡ | | 6.49 ± 1.06§ |
| T Cholesterol (mg/dL) | 209.30 ± 31.37 | 178.67 ± 22.27 | 189.46 ± 35.10* | | 150.67 ± 25.18§§ |
| LDL (mg/dL) | 135.60 ± 27.75 | 111.80 ± 19.14 | 119.62 ± 31.54* | | 86.00 ± 20.58§§ |
| HDL (mg/dL) | 57.70 ± 14.44†† | 51.20 ± 11.60 | 44.54 ± 12.12‡ | | 43.67 ± 8.60 |
| TG (mg/dL) | 115.80 ± 42.98† | 122.67 ± 47.80 | 258.00 ± 240.29 | | 159.00 ± 65.02 |
| Plasma Cr (mg/dL) | 0.95 ± 0.39 | 0.82 ± 0.15 | 0.98 ± 0.46 | | 0.99 ± 0.28 |
| GFR (mL/min/1.73 m^2^) | 89.89 ± 33.96 | 89.42 ± 15.91 | 90.85 ± 33.21 | | 82.81 ± 23.51 |
| Ratio UAE/Cr (mg/g) | 249.12 ± 274.62† | 5.39 ± 7.33 | 352.58 ± 305.09 ** | | 3.03 ± 1.48 |

BMI: body mass index; Cr: creatinine; DBP: diastolic blood pressure; GFR: glomerular filtration rate; Hb: hemoglobin; HDL: high density lipoprotein; LDL: low density lipoprotein; SBP: systolic blood pressure; T Cholesterol: total cholesterol; TG: triglycerides; UAE: urinary albumin excretion. Comparisons between diabetic groups: * *p* < 0.05, ** *p* < 0.001. Comparisons between diabetic and non-diabetic groups: † *p* < 0.05; †† *p* < 0.01; ††† *p* < 0.001. Comparisons between increased UAE groups: ‡ *p* < 0.05, ‡‡ *p* < 0.01, ‡‡‡ *p* < 0.001. Comparisons between No UAE groups: § *p* < 0.05, §§ *p* < 0.01.
